# Fluid volume expansion and depletion in hemodialysis patients lack association with clinical parameters

**DOI:** 10.1186/s40697-015-0090-5

**Published:** 2015-12-22

**Authors:** Sylvia Kalainy, Ryan Reid, Kailash Jindal, Neesh Pannu, Branko Braam

**Affiliations:** Department of Medicine, Division of Nephrology, University of Alberta, 11-107 CSB, Edmonton, AB T6G 2G3 Canada; Department Physiology, University of Alberta, Edmonton, Canada

**Keywords:** Hemodialysis, Extracellular fluid volume, Hypertension, Bio-impedance

## Abstract

**Background:**

Achievement of normal volume status is crucial in hemodialysis (HD), since both volume expansion and volume contraction have been associated with adverse outcome and events.

**Objectives:**

The objectives of this study are to assess the prevalence of fluid volume expansion and depletion and to identify the best clinical parameter or set of parameters that can predict fluid volume expansion in HD patients.

**Design:**

This study is cross-sectional.

**Setting:**

This study was conducted in three hemodialysis units.

**Patients:**

In this study, there are 194 HD patients.

**Methods:**

Volume status was assessed by multifrequency bio-impedance spectroscopy (The Body Composition Monitor, Fresenius) prior to the mid-week HD session.

**Results:**

Of all patients, 48 % (*n* = 94) were volume-expanded and 9 % of patients were volume-depleted (*n* = 17). Interdialytic weight gain was not different between hypovolemic, normovolemic, and hypervolemic patients. Fifty percent of the volume-expanded patients were hypertensive. Paradoxical hypertension was very common (31 % of all patients); its incidence was not different between patient groups. Intradialytic hypotension was relatively common and was more frequent among hypovolemic patients. Multivariate regression analysis identified only four predictors for volume expansion (edema, lower BMI, higher SBP, and smoking). None of these parameters displayed both a good sensitivity and specificity.

**Limitations:**

The volume assessment was performed once.

**Conclusions:**

The study indicates that volume expansion is highly prevalent in HD population and could not be identified using clinical parameters alone. No clinical parameters were identified that could reliably predict volume status. This study shows that bio-impedance can assist to determine volume status. Volume status, in turn, is not related to intradialytic weight gain and is unable to explain the high incidence of paradoxical hypertension.

## Abrégé

**Mise en contexte:**

L’atteinte d’une volémie normale est primordiale dans le suivi des patients sous hémodialyse. Les états d’expansion et de contraction du volume hydrique sont associés aux complications majeures de l’insuffisance rénale et à une issue défavorable de la maladie.

**Objectifs de l’étude:**

Cette étude visait à évaluer la prévalence de l’expansion et de la déplétion des fluides en situation d’hémodialyse. On a également voulu identifier les paramètres cliniques qui pourraient permettre de prévoir les épisodes d’expansion de volume hydrique chez les patients dialysés.

**Cadre et type d’étude:**

Il s’agit d’une étude transversale qui s’est tenue dans trois unités d’hémodialyse, sur un total de 194 patients.

**Methodes:**

La volémie des Par spectroscopie de bio-impédance à multifréquence (« Body composition Monitor » de Fresenius) tout juste avant la séance d’hémodialyse prévue en milieu de semaine.

**Résultats:**

De tous les patients qui ont participé à l’étude, une proportion de 48 % (n = 94) était en situation d’expansion volumique alors que 9 % (n = 17) se trouvaient en état de déplétion. Toutefois, la prise de poids interdialytique était semblable pour tous les patients, qu’ils aient été hypovolémiques, normovolémiques ou hypervolémiques avant la séance d’hémodialyse. Il est à noter que la moitié des patients (50 %) en situation d’expansion volémique souffraient également d’hypertension artérielle. De fait, l’hypertension artérielle paradoxale s’est avérée répandue parmi les patients dialysés (31 %), mais aucune variation notable de son incidence n’a été observée dans les différents groupes. Les cas d’hypotension intradialytiques quant à eux, se sont avérés relativement courants, particulièrement chez les sujets hypovolémiques avant la séance d’hémodialyse. L’analyse par régression multivariée n’a révélé que quatre indicateurs susceptibles d’aider à prévoir les épisodes d’expansion de volume : la présence d’œdème, un indice de masse corporelle faible, une pression artérielle systolique élevée avant l’hémodialyse, ainsi que le fait de fumer. Cependant, aucun de ces paramètres n’a présenté une spécificité au plan diagnostique ni une sensibilité particulière pour prévoir les épisodes d’expansion de volume.

**Limites de l’étude:**

La volémie des participants n’a été mesurée qu’une seule fois lors de cette étude.

**Conclusion:**

L’étude a révélé une prévalence élevée de l’expansion du volume hydrique chez les patients sous hémodialyse, mais il a été impossible de la détecter à l’aide des paramètres cliniques utilisés. Qui plus est, aucun des paramètres mesurés n’a permis d’anticiper avec certitude des variations de la volémie. Concernant la bio-impédance, les résultats ont révélé qu’elle pourrait aider à évaluer l’expansion de volume chez les patients dialysés. En revanche, cette dernière ne peut être associée directement à la prise de poids intradialytique pas plus qu’elle ne peut à elle seule expliquer la forte incidence d’hypertension artérielle paradoxale.

## What was known before

Prevalence of extracellular fluid volume expansion has been previously reported to be high.

## What this adds

The present study confirms the high prevalence of volume expansion and indicates that this cannot be estimated by clinical parameters. Moreover, the study adds that volume contraction is also relatively common. Last, volume status is not related to intradialytic weight gain and is unable to explain the high incidence of paradoxical hypertension.

## Background

Accurate assessment of volume status (VS) remains one of the greatest challenges in the treatment of hemodialysis (HD) patients [1]. Chronic volume expansion contributes to hypertension, left ventricular hypertrophy, and heart failure in HD patients [2–6]. Conversely, volume contraction could predispose the HD patient to intradialytic hypotension, cramps, arrhythmias, cardiac stunning, and reduced well-being after treatment [2]. Therefore, adequate extracellular fluid volume (ECFV) control is crucial for blood pressure regulation and for prevention of cardiovascular complications in this population [7].

Clinical assessment of volume expansion by assessing blood pressure, edema, and central venous pressure has limitations [8–11]. Interdialytic weight gain (IDWG) is not sufficient to assess volume expansion since it does not necessarily correlate with ECFV expansion [12]. The inferior vena cava collapse index [2], ultrasound assisted assessment of pulmonary fluid content [13], and echocardiography [2] are helpful to assess volume status, but they do not provide an estimate of total ECFV expansion or depletion and are impractical for usage in clinical practice. Multifrequency bio-impedance spectroscopy is a convenient method to assess extracellular and intracellular fluid volume [14]. This method has been validated against gold standard dilution methods [14, 15] and is applicable in the setting of HD [16].

Given the mortality associated with ECFV expansion [6, 17] and the knowledge that adequate volume control provides better control of blood pressure [18], more accurate objective methods for volume assessment would be valuable. In the current study, we tested the hypothesis that volume expansion is highly prevalent in HD patients, likely due to the inability to judge volume status from clinical parameters. Bio-impedance spectroscopy was applied to assess volume status in an in-center HD population and compared with clinical volume assessment. Our aims were (1) to assess the prevalence of volume expansion and volume depletion in our HD population using bio-impedance spectroscopy measurements, (2) to investigate the association between clinical parameters and volume status as assessed by bio-impedance, and (3) to search for a set of clinical parameters that best predict volume status in HD patients.

## Methods

### Patients

Two hundred HD sequential adult patients were included in the study. Inclusion criteria included all prevalent HD patients who agreed to participate in the study in three HD units of the Northern Alberta Renal Program. Patients were not included or excluded based on their blood pressure. Exclusion criteria were as follows: patients with a pacemaker or implanted defibrillator, amputation, and metallic prosthesis. Six patients were excluded from the analysis since they were new hemodialysis starts (<6 weeks) before the measurement. Ethics approval was obtained from the Human Research Ethics Board at the University of Alberta Hospital. All patients included in the study provided written informed consent.

### Evaluation of volume status

The Body Composition Monitor (BCM, Fresenius Medical Care, Bad Homburg, Germany) is a multifrequency bio-impedance device that provides a convenient method to obtain extracellular fluid volume (ECFV) and has been validated previously [19]. The BCM uses the same technology as the HYDRA ECF/ICF 4200 platform (Xitron, San Diego, USA) used in this validation study. Overhydration is calculated by comparison of the measured ECFV with the predicted ECFV using a physiological model [20] with adjustments for subjects with high body mass index [21]. Measurements were performed at one occasion in triplicate with the device. Measurements were performed before the start of mid-week HD treatment with the patients in supine position for 10 min. Electrodes were applied on the ipsilateral arm and foot of the non-AV-fistula side. The BCM measures the impedance of different body compartments at 50 different frequencies between 5 and 1000 kHz. The BCM calculates volume status (VS) which is expressed as volume excess or depletion in liters compared to the estimated normal ECFV. The accuracy of bio-impedance in ECFV is estimated to be within −0.4 ± 1.4 L when compared to dilution methods [19]. To facilitate comparison between patients, the volume status was normalized to estimated extracellular fluid volume (VS/ECFV). The patient population was divided into hypovolemic, normovolemic, and hypervolemic groups. Volume contraction was considered more than 7 % below normal ECFV (equivalent to 1.1 L below normal ECFV) prior the mid-week HD session. Normovolemia was considered any measurement between −7 and +7 % relative to normal ECFV. Volume expansion was considered more than 7 % above normal ECFV (equivalent to 1.1 L above normal ECFV). The 7 % cutoff point was based on the thought that with a 75 mmol/d sodium intake, patients ideally swing from −1.1 below normal ECFV after their run to +1.1 L above normal ECFV before the next run.

### Clinical and biochemical parameters

Clinical parameters collected include pre- and post-dialysis blood pressure for the same session and five previous sessions. BP was measured in the sitting position. Hypertension was considered if the average pre-dialysis BP exceeded 140/90 mmHg for the current and the four previous sessions or if the patient had a history of hypertension as defined by being prescribed antihypertensive medication. The prevalence of hypertension was calculated from this composite data. Intradialytic hypotension was defined as post-dialysis SBP falling below 100 mmHg and the difference between pre-dialysis SBP and post-dialysis SBP >20 mmHg with accompanying clinical symptoms during dialysis that required an intervention or cessation of ultrafiltration [22]. As there is no widely accepted definition of paradoxical hypertension [23], we considered it as a rise of SBP of >20 mmHg during or after dialysis with post-dialysis BP exceeding 140/90 mmHg. Patients were considered to have diabetes if it was mentioned in their charts or if the patient was on anti-diabetic medications. Pedal edema was assessed as present or absent. Dry weight (DW) was obtained from the patient charts. IDWG for the previous five sessions was recorded. IDWG was calculated by subtracting the post-dialysis weight of previous HD session from the pre-dialysis weight of the index HD session. To determine the correlation between IDWG and volume expansion, IDWG more than 7 % of ideal ECFV was considered elevated. All biochemical parameters (plasma Na, K, serum albumin, WBCs, urea reduction ratio, and cholesterol level) were obtained from the most recent monthly blood work of the patient. Clinical volume assessment was performed weekly by rounding physicians. Dialysate sodium composition was 137 mmol/L for all patients regardless of their plasma sodium concentration.

### Statistical analysis

Continuous data are expressed as mean ± standard deviation. Categorical variables are expressed as percentage of total. One way ANOVA was used for univariate comparisons. Bonferroni multiple comparison test was used to detect between group differences when ANOVA showed a statistically significant result. Pearson’s test was used for univariate correlations. Multiple linear regression was performed with VS/ECFV% (volume status/extracellular fluid volume) as the target variable, to find predictors for volume expansion. Variables selected for the multivariate model based on a significant univariate analysis with a *P* value <0.10. Sensitivity and specificity were calculated using standard formulae. All data analysis was done with Graph prism (Graphpad 5, San Diego, CA, USA) and SPSS version 21 (SPSS Inc., Chicago, IL, USA). A *P* value <0.05 was considered statistically significant.

## Results

### General characteristics of the study population

Forty-five percent of the 194 participants were hypertensive as classified by the average pre-dialysis blood pressure for five HD sessions. On average patients displayed volume expansion with an average volume status of +7.8 % (volume expansion related to ECFV). Antihypertensive medication was prescribed in 48 % of patients. Most commonly prescribed were beta-blockers (26 %), calcium channel blockers (21 %), and angiotensin-converting enzyme inhibitors (12 %) Angiotensin receptor blockers and loop diuretics were prescribed in 3 and 14 % of patients, respectively. Characteristics of the patients are displayed in Table 1.

**Table 1:** Characteristics of the study population

Characteristics	All patients (*n* = 194)	Hypovolemic (*n* = 17)	Normovolemic (*n* = 83)	Hypervolemic (*n* = 94)	*P* value
VS, L (assessed by BCM)	1.1 ± 2	−2.1 ± 0.6	0.1 ± 0.7^a^	2.6 ± 1.5^b,c^	<0.001*
VS/ECFV% (assessed by BCM)	7.8 ± 12	−12 ± 3.4	0.9 ± 4^a^	17 ± 10^b,c^	<0.001*
Clinical target weight	72 ± 22	85 ± 28	74 ± 21	69 ± 20^c^	0.014*
VS, L (clinically assessed)	2.2 ± 2.0	2.1 ± 1.3	1.7 ± 1.5	2.5 ± 2.3^b^	0.016*
VS/ECFV (clinically assessed)	0.2 + 1.2	11.6 ± 8.2	10 ± 9	16.8 ± 15^b^	0.001*
Gender, M/F	115/79	10/7	47/36	58/36	0.702
Age, years	61 ± 15	60 ± 16	60 ± 16	62 ± 15	0.788
Diabetes, %	45 %	47 %	3 %	54 %^c^	0.035*
Smoking, %	11 %	0 %	6 %	17 %^c^	0.02*
Edema, %	28 %	0 %	9 %	47 %^b,c^	<0.001*
Obesity, %	26 %	47 %	30 %	19 %^b,c^	0.001*
Pre-HD-SBP, mmHg	131 ± 25	128 ± 26	129 ± 26	137 ± 25	0.088
Pre-HD-DBP, mmHg	71 ± 16	70 ± 15	72 ± 18	72 ± 16	0.843
Pre-HD-PP, mmHg	60 ± 22	59 ± 29 ^b^	57 ± 19	65 ± 19^b^	0.025*
Pre-HD-MAP, mmHg	91 ± 17	89 ± 14	91 ± 19	94 ± 17	0.418
HTN, %	45 %	41 %	36 %	54 %^c^	0.015*
Antihypertensive	48 %	24 %	45 %	54 %	0.057
Intradialytic hypotension	17 %	35 %	20 %	11 %^b^	0.007*
Paradoxical hypertension	31 %	35 %	29 %	32 %	0.840
Plasma sodium, mmol/L	136 ± 3	136 ± 2.6	137 ± 3	135.5 ± 3^c^	0.002*
Serum potassium, mEq/L	4.7 ± 0.6	4.8 ± 0.6	4.6 ± 0.6	4.8 ± 0.7	0.163
Albumin, g/L	36 ± 3.5	36 ± 4	37 ± 3.	36 ± 3.5	0.184

*VS* volume status, *VS/ECFV* volume status/extracellular fluid volume, *pre-HD-SBP* pre-hemodialysis systolic blood pressure, *pre-HD-DBP* pre-hemodialysis diastolic blood pressure, *pre-HD-PP* pre-hemodialysis pulse pressure, *pre-HD-MAP* pre-hemodialysis mean arterial pressure, *HTN* hypertension

*P < 0.05

^a^Significant difference between hypovolemic and normovolemic

^b^Significant difference between hypovolemic and hypervolemic

^c^Significant difference between normovolemic and hypervolemic

### Prevalence of volume abnormalities

**Fig. 1:**
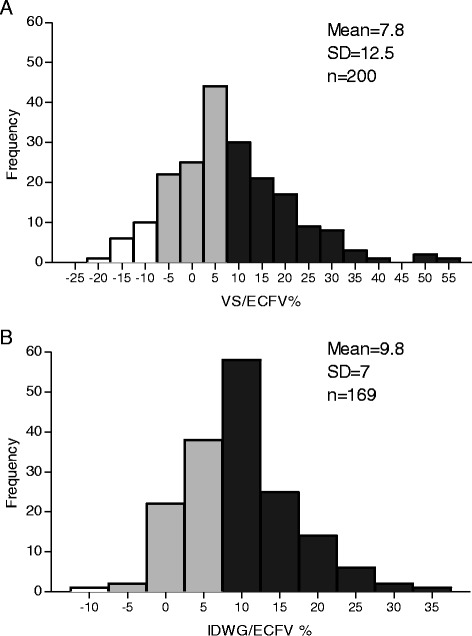
**a** Frequency distribution of volume status corrected for extracellular fluid volume (VS/ECFV) for the whole study population. **b** Frequency distribution of interdialytic weight gain (IDWG) for the whole study population corrected for ECFV. *VS/ECFV* volume status/extracellular fluid volume, *IDWG/ECFV* interdialytic weight gain/extracellular fluid volume

VS of all patients is shown in Fig. 1. In 43 % of the patients VS was normal (between −7 % and +7 % of the ideal ECFV); 48 % percent of all patients displayed >7 % ECFV expansion. Of these, 47 % (23 % of all patients) exhibited volume expansion >15 % of ECFV. Volume contraction of >7 % was observed in 9 % of the patients. Interestingly, clinically assessed fluid overload (in percentage of normal ECFV) in the hypervolemic group was significantly higher than the normovolemic group but not different from the hypovolemic group. Clinically assessed volume status and volume status assessed by the BCM were correlated, yet the slope was 0.265 and patients were estimated to be 11 % fluid overload clinically at the point where the BCM did not detect any fluid overload.

### Comparison of characteristics of hypovolemic, normovolemic, and hypervolemic patients

**Fig. 2:**
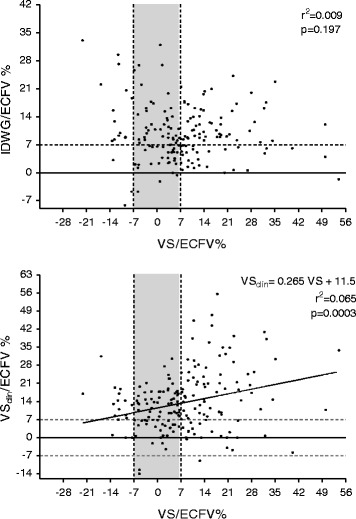
**a** No significant correlation between volume status and IDWG (both corrected for ECFV) could be demonstrated, *P* = 0.985. **b** Correlation between clinically assessed volume status (VS_clin_/ECFV) and volume status assessed with the BCM (VS/ECFV). *IDWG/ECFV* interdialytic weight gain/extracellular fluid volume, *VS/ECFV* volume status/extracellular fluid volume as assessed by the BCM, *VS*_*clin*_ */ECFV* volume status as clinically assessed/extracellular fluid volume

Hypertension, diabetes, smoking, and edema were more common and intradialytic hypotension less common in hypervolemic patients. Pulse pressure was higher in hypervolemic patients. When patients with mild volume expansion (+7 % < VS/ECFV < +15 %) were compared to patients with severe volume expansion (VS/ECFV > +15 %), incidence of edema and plasma potassium was higher in patients with severe volume expansion. No other clinical parameters were different between these patients. IDWG/ECFV displayed wide variation yet IDWG/ECFV and VS/ECFV were not correlated (Fig. 2; *r*^2^ = 0.009945, *P* = 0.19).

### Relation between blood pressure and volume status as assessed by bio-impedance

Twenty eight percent of the patients had hypertension and ECFV expansion. Of all patients, 23.5 % were normotensive and normovolemic and 19.5 % had normal blood pressure despite volume expansion. The majority of volume-expanded patients had normal blood pressure. Pre-dialysis BP was not significantly different between groups. Only Pre-dialysis PP was significantly higher in normovolemic patients compared to hypovolemic patients. Only pre-dialysis SBP showed a weak correlation to VS corrected for ECFV.

### Incidence of intradialytic hypotension and paradoxical hypertension

**Fig. 3:**
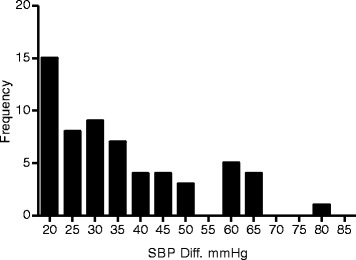
Frequency distribution of severity of paradoxical hypertension, illustrated as rise in SBP in mmHg (post-dialysis SBP—pre-dialysis SBP), average rise of SBP from pre- to post-dialysis was 38 mmHg. *SBP* systolic blood pressure

Intradialytic hypotension was found in 17 % of patients. Intradialytic hypotension was significantly more common in hypovolemic patients (*P* = 0.007). The incidence of paradoxical hypertension was high (31 %), yet prevalence was not different between groups. The distribution of severity of paradoxical hypertension is shown in Fig. 3.

### Clinical parameters and volume expansion

After univariate analysis, nine variables were included in the multiple regression model (DM, HTN, edema, BMI, smoking, pre-HD-PP, pre-HD-SBP, sodium, and albumin). Edema, lower BMI, SBP, and smoking were significant predictors for volume expansion (Table 2). Relative risk was most pronounced for edema (Table 3). Sensitivity, specificity, and positive and negative predictive values for the four individual parameters are shown in Table 3 and precluded the development of a robust volume expansion score. The cutoff points for calculating sensitivity and specificity for continuous variables were 140 mmHg for SBP and 25 for BMI, respectively.

**Table 2:** Results of the multivariate analysis (with VS/ECFV % as the target variable)

Parameter	Unstandardized Coefficient B	95 % CI		*r*	*P* value
		Lower bound	Upper bound		
DM	2.71	–0.37	5.80	0.15	0.084
Smoking	4.91	0.693	9.12	0.12	0.037*
Edema	12.82	9.58	16.06	0.48	<0.0001*
Obesity	–0.66	–0.87	–0.44	–0.27	<0.0001*
Pre-HD-SBP, by 1 mmHg	0.092	–0.011	0.19	0.34	0.001*
Pre-HD-PP, by 1 mmHg	0.004	–0.103	0.11	0.31	0.94
HTN	0.33	–3.07	3.73	0.16	0.85
Plasma Sodium,, by 1 mmol/L	0.34	–0.12	0.80	–0.07	0.15
Albumin, by 1 g/L	–0.21	–0.64	0.21	–0.11	0.32

*DM* diabetes mellitus, *pre-HD-SBP* pre-hemodialysis systolic blood pressure, *pre-HD-PP* pre-hemodialysis pulse pressure, *HTN* hypertension

*P < 0.05

**Table 3:** Sensitivity and specificity of single clinical parameters in predicting volume expansion

Criteria	Relative risk	PPV (%)	NPV (%)	Sensitivity (%)	Specificity (%)
Edema	2.4	85	65	47	92
Lower BMI	1.6	54	67	84	32
Pre-HD-SBP	1.5	60	60	55	64
Smoking	1.5	73	54	16	94

*BMI* body mass index, *pre-HD-SBP* pre-hemodialysis systolic blood pressure

## Discussion

In the current study, ECFV expansion as assessed by bio-impedance in in-center HD patients was highly prevalent. ECFV contraction was also observed and was more frequently associated with intradialytic hypotension. Paradoxical hypertension was highly prevalent and not associated with volume status. Several clinical parameters were more prevalent in HD patients with ECFV expansion, however, were not sufficiently sensitive and specific to be applied to robustly assess volume status.

Fluid volume expansion in HD patients is a well-recognized problem [12, 24] associated with hypertension [2, 3], left ventricular hypertrophy, dilated cardiomyopathy [4], heart failure, and eventually with high mortality [5, 6]. Strikingly, about 50 % of our patients displayed mild to severe ECFV expansion of >7 % of ECFV. Moderate to severe ECFV expansion of >15 % of ECFV was observed in 23 % of the patients, despite frequent routine clinical volume assessment. Previous studies using bio-impedance for quantification of volume status reported slightly lower [25] or similarly high prevalence of ECFV expansion [4, 5]. Of note, volume status and interdialytic weight gain were not correlated in the current study. This implies that strategies to improve volume status need to separately address dry weight and dietary salt intake to achieve good volume regulation. Underscoring the relevance of IDWG, one study reported that reducing the interdialytic weight gain without changing the dry weight reduced ventricular hypertrophy [26]. Therefore, although the debate as to whether chronic ECFV expansion or IDWG is the most important factor determining cardiovascular outcome is not settled, controlling fluid overload can reduce hypertension and LVH and improve outcome [27]; controlling the latter could decrease cardiac stunning [28, 29].

Pre-dialysis volume depletion of >7 % of ECFV (comparable to 1.1 L for average ECFV) was demonstrated in 9 % of patients, compared to >1.1 L in 5 % of patients in a previous report [25]. Volume-depleted patients only differed from normo- or hypervolemic patients regarding more frequent intradialytic hypotension. Intradialytic hypotension occurred in 17 % of all patients. Previous studies reported the incidence of intradialytic hypotension to be between 15 and 25 % of HD sessions [30, 31]. Since volume status was assessed pre-dialysis and several hypovolemic patients had substantial interdialytic weight gains of >25 % of ECFV, these patients would be severely hypovolemic post-dialysis. Although several patients with volume depletion had very substantial interdialytic weight gain, no correlation could be established between interdialytic weight gain and volume contraction. Fluid overload has previously been inversely associated with IDWG, in about 15000 patients from 60 dialysis centers [12]. Taken together, our study indicates that a relevant fraction of patients showed fluid depletion and a higher risk of intradialytic hypotension.

Hypertension was present in 54 % (27 % of total) of hypervolemic and in 36 % (19 % of total) of normovolemic patients. Fluid status and systolic blood pressure were correlated, in contrast to ECFV expansion and diastolic and pulse pressure. This shows that the relationship between blood pressure and volume status is complex. Wabel et al. analyzed the relation between blood pressure and volume expansion using comparable methodology in 500 HD patients [32]. Volume-dependent hypertension was found in 15 % of patients; the majority of patients (27 %) were normotensive and normovolemic. Only 10 % of patients had normal blood pressure despite volume expansion. This suggests that volume expansion is responsible for hypertension in HD patients; yet, the use of antihypertensives might obscure the importance of fluid overload for BP control.

Paradoxical hypertension in HD patients has been associated with increased risk of mortality [33]. Paradoxical hypertension was common in our study population (31 %) and was not significantly different among the three patient groups. Limited information is available about the prevalence of paradoxical hypertension and its relation to volume status in a large HD population. A previous study using similar definition of paradoxical hypertension reported prevalence in 21 % of patients [34]. UF rate was significantly lower in patients with paradoxical hypertension; however, no assessment of ECFV was performed [34]. Using several different definitions for paradoxical hypertension, other studies report incidence of 10–15 % [23]. A recent study reported a decline in the incidence of paradoxical hypertension with excessive ultrafiltration concluding that intradialytic hypertension may be a sign of volume expansion [35]; ECFV was not assessed in that study. Altogether, our data indicate a high frequency of paradoxical hypertension but no clear association with volume expansion. Moreover, it underscores the need to understand this important problem with consequences for outcome better [36].

In search for clinical and laboratory characteristics associated with volume status, multivariate regression analysis identified only four predictors for volume expansion (edema, lower BMI, higher SBP, and smoking). None of these parameters displayed both a good sensitivity and specificity. In our study, the presence of edema had a good positive predictive value. A previous study reported that pedal edema correlates well with cardiovascular risk factors and left ventricular mass, but it did not reflect volume in HD patients as assessed by cardiac biomarkers and echocardiography [37]. Using a similar methodology in PD patients, multiple regression analysis revealed that DM, higher SBP, older age, male gender, lower serum albumin, and lower BMI were significant predictors for volume expansion [38]. It seems therefore that a set of parameters that can assist to better assess fluid volume in HD patients on clinical grounds cannot be formulated. In addition to the evaluation of specific clinical indicators of fluid overload, we related the clinically assessed volume status to the estimated normal ECFV and compared this with the volume status measured by the BCM. This yields the interesting observation that although there is a positive correlation between clinically assessed and BCM assessed volume status, clinical assessment indicated fluid overload at normovolemia assessed by BCM. Moreover, the slope between clinically assessed and BCM assessed VS was far below unity. This indicates that a clinical assessment is not precise and that severe fluid overload is underestimated.

Our study has several limitations. First of all, bio-impedance spectroscopy-based extracellular fluid volume assessment has an error of −0.4 ± 1.4 L compared to gold standard dilution methods [19]. The study did not separately assess the validity on the 120 healthy subjects in the study and the 32 HD patients. However, a recent review reported that radioisotope methods, previously considered the gold standard, have similar accuracy compared to bio-impedance in dialysis patients [39]. Moreover, bio-impedance is highly reproducible with inter-observer and intra-observer errors of less than 2 % [39]. The measurement was performed once, prior to the second dialysis session of the week. Although this could introduce some noise, it is not likely that it would affect our interpretation of the data. Finally, we cannot assure that different devices to assess fluid status and different methodology to calculate the normal ECFV or an individual would not lead to slightly different numerical results. It is considered not likely that the overall interpretation of the study would have been different.

## Conclusions

In summary, using bio-impedance spectroscopy, we found that volume expansion is highly prevalent in our HD patients and volume contraction was also not uncommon. Intradialytic hypotension was more common in hypovolemic patients. Fluid volume expansion or contraction could not be reliably identified by clinical parameters, except that edema predicted fluid volume expansion. This study shows that bio-impedance can assist to determine volume status. Volume status, in turn, is not related to intradialytic weight gain and is unable to explain the high incidence of paradoxical hypertension. Whether volume expansion or contraction determined using bio-impedance is associated with worse outcome still needs to be determined.
